# The relationship between subjective oral frailty and adverse health outcomes or medical and dental expenditures in the latter‐stage older adult: A 6‐year longitudinal study

**DOI:** 10.1002/cre2.717

**Published:** 2023-02-05

**Authors:** Tokiko Doi, Makoto Fukui, Masami Yoshioka, Yoshifumi Okamoto, Manabu Shimomura, Kimi Matsumoto, Miwa Matsuyama, Daisuke Hinode

**Affiliations:** ^1^ Department of Hygiene and Oral Health Science Tokushima University Graduate School of Biomedical Sciences Tokushima Japan; ^2^ Faculty of Health and Welfare Tokushima Bunri University Tokushima Japan; ^3^ Tokushima Prefecture Dental Association Tokushima Japan; ^4^ Department of Oral Health Care and Rehabilitation Tokushima University Graduate School of Biomedical Sciences Tokushima Japan

**Keywords:** adverse health outcome, longitudinal study, medical expenditures, oral frailty

## Abstract

**Objectives:**

The study aimed to investigate the relationship between subjective oral frailty and adverse health outcomes or medical and dental expenditures in the latter‐stage older adult through a 6‐year longitudinal study.

**Methods:**

The participants enrolled in the cross‐sectional study were 3564 Tokushima City residents aged 75, 80, 85, and 90 years old who received oral health examinations and who responded to a questionnaire on oral conditions and health behavior at baseline. The data for the occurrence of disability or mortality, and the monthly medical expenditures, dental expenditures, and total medical expenditure of each participant were obtained from the National Health Insurance Database.

**Results:**

The total medical expenditure showed significant differences in participants who had difficulties in eating tough foods, difficulties in swallowing tea or soup, and dry mouth when compared to that of healthy participants, in addition to the current medical treatment against the general disease. The 6‐year longitudinal study revealed that participants with subjective oral frailty symptoms, including difficulties in eating tough foods and difficulties in swallowing tea or soup at baseline, had significantly higher medical, dental, and total expenditures among 538 participants without certified nursing care. In addition, those with subjective oral frailty or with less than 19 teeth present were shown to have a higher possibility for the occurrence of disability or mortality by the cox proportional hazard analysis. Furthermore, it was found that medical and total expenditures in older adults with adverse health outcomes were higher than that of healthy participants.

**Conclusion:**

These results suggest that subjective oral frailty in the latter‐stage older adult is related to subsequent adverse health outcomes and an increase in medical and dental expenditures.

## 1 INTRODUCTION

Japan is aging at a speed unprecedented in other countries; the proportion of older adults aged 75 and over in the total population is expected to exceed 25% in 2055 (Ministry of Health Labor and Welfare, 2012). The increase in medical care expenditures for older adults has become a serious problem under the current circumstances, and the medical expenditures for older adults account for more than half of the national medical expenditures in the current situation (Ministry of Health Labour and Welfare, 2011). The need to extend healthy life expectancy has been promoted as one of the measures to reduce medical expenditures. In 2019, the “Healthy Life Expectancy Extension Plan” was formulated in Japan: the healthy life expectancy will be extended by more than 3 years for both men and women, aiming to reach 75 years or older by 2040. To achieve this plan, three fields, including care prevention, frailty measures, and dementia prevention will be promoted (Ministry of Health Labour and Welfare, 2020). Therefore, it is important to focus on frailty prevention.

In the field of dentistry, the concept of “oral frailty,” in which oral function is weakened, was proposed in 2015. It reported that the period of pre‐frailty begins with a decline in social activities such as loneliness and a decline in oral health literacy, and then the second stage is oral frailty with a slight decline in oral function (decreased movement of the tongue to pronounce, spilled food, difficulties in chewing well, etc.) (Iijima, 2017). It also emphasized that the oral frailty period was the entrance to the frailty period of the body and is a critical period that gradually shifts to the irreversible frailty period if it is overlooked (Iijima, 2017).

It has been reported that six oral indicators are related to oral frailty: the number of natural teeth, chewing ability, articulatory oral motor skill, and subjective difficulties in eating and swallowing (Tanaka et al., 2018), and participants with poor oral status have been significantly associated with the risk of physical frailty, sarcopenia, disability, and mortality when compared to healthy participants (Tanaka et al., 2018). It was also revealed in our previous study that oral frailty‐related symptoms such as “difficulty eating tough foods” predicted the onset of adverse health outcomes including disability or mortality in 75‐year‐old persons (Sahara et al., 2022).

The national government in Japan is promoting the implementation of oral health examinations for residents aged 75 and over to prevent the deterioration of oral function and prevent diseases such as pneumonia (Ministry of Health Labour and Welfare, 2016). It was reported that regular dental checkups lead to decreased dental expenses (Moeller et al., 2010). The promotion of oral health measures and the reduction of medical expenditures for older adults are urgent issues in Japan. It showed that subjective assessment of oral health was significantly related to medical expenditure in older adults (Sato et al., 2016).

The aim of this study was to investigate the relationship between subjective oral frailty symptoms and medical and dental expenditures in latter‐stage older adults using data obtained from the oral health examination and the national health insurance through a 6‐year longitudinal study. In addition, the association between oral frailty and the occurrence of adverse health outcomes, such as disability and mortality, was analyzed.

## 2 MATERIALS AND METHODS

### 2.1 The cross‐sectional study

Tokushima Association Responsible for the Operation of the Health‐Care System for Latter‐Stage Older Adults conducted the “Oral health examination for latter‐stage older adults” program for residents aged 75, 80, 85, and 90 in Tokushima Prefecture as a national treasury subsidy project, in collaboration with the Tokushima Dental Association. The participants who received an oral health examination ticket were able to have a free examination at the dental clinic. Dentists participating in the oral health examination project were required to attend a training session conducted by Tokushima Dental Association.

**Figure 1:**
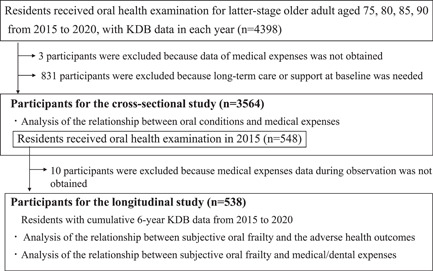
Flow diagram of this study.

As shown in Figure 1, the participants included in this study were 4398 older adult residents of Tokushima City who underwent oral health examinations for latter‐stage older adults from 2015 to 2020. The number of dental clinics where the participants visited and received examinations was 229. Three participants whose data on medical expenditures was not obtained and 831 participants who were certified as requiring long‐term care or support at the baseline were excluded. Finally, 3564 participants were enrolled in the cross‐sectional study.

The results of the questionnaire survey and the data on oral health status, which was obtained from “Oral health examination for latter‐stage older adults” from 2015 to 2020, were used in this study. The monthly medical expenditures, dental expenditures, and total expenditure of each participant were also obtained from the National Health Insurance Database (KDB), and the annual cumulative expenditures were then calculated. The Tokushima Association Responsible for the Operation of the Health‐Care System for the Latter‐Stage Older Adults collated the data in a form that did not identify individuals, then provided those as research data. The relationship between the oral health behavior/the oral condition and the annual cumulative expenditures in a single year was investigated for the oral health factors involved in medical expenditure.

### 2.2 The longitudinal study

Among 3564 participants, 548 received oral health examinations for latter‐stage older adults in 2015. Ten were eventually excluded because no data was obtained during the 6‐year observation after they moved out of the prefecture. Thus, 538 participants were enrolled in the longitudinal study. The results of the questionnaire survey and oral conditions from the results of oral health examination data in 2015 were used as baseline data. In addition, the cumulative 6‐year (from 2015 to 2020) medical expenditures, dental expenditures, and total expenditure of each individual in the KDB were calculated and used for analyzing the relationship with the oral health behavior or the oral condition at baseline. The oral health factors involved in medical expenditure were investigated as well in the longitudinal study. In addition, the data on adverse health outcomes, which was the occurrence of disability (support/long‐term care certification) or mortality in each month of the KDB, was also obtained to analyze the association with the oral condition at baseline.

### 2.3 Items of the questionnaire survey

Among the questionnaire survey items from 2015 to 2020, the items that could be evaluated without changing the contents of the questionnaire were tabulated as shown in Table 1. Gender, age (75, 80, 85, or 90 years old), history of pneumonia within 1 year (presence/absence), current medical treatment of diabetes, stroke, heart disease, and cancer (presence/absence), “Is it difficult to eat tough foods than half a year ago?”: difficulties in eating tough foods (yes/no), “Is it difficult to swallow tea or soup?”: difficulties in swallowing tea or soup (yes/no), were set as survey items. In this study, the items of difficulties in eating tough foods and difficulties in swallowing tea or soup, corresponding to either one or both were regarded as “subjective oral frailty symptoms” and used for analyzing items. Furthermore, body mass index (BMI) was calculated from the height and weight obtained in the questionnaire, and participants were categorized as “thin” if their BMI was less than 21.5.

**Table 1:** The difference in medical, dental, and total expenditures according to items and categories in the questionnaire.

						Medical expenditure^a^			Dental expenditure^a^			Total expenditure^a^		
Items		Category	*n*	(%)		Mean	SD	*p*‐value^b^	Mean	SD	*p*‐value^b^	Mean	SD	*p*‐value^b^
Questionnaire survey														
		75‐year‐old	1917	(53.8)		12.3	1.9	**	9.4	3.5	**	12.6	1.1	**
	Age	80‐year‐old	1123	(31.5)		12.4	1.7		9.1	3.9		12.8	1.0	
		85‐year‐old	443	(12.4)		12.7	1.4		8.9	4.0		12.9	0.8	
		90‐year‐old	81	(2.3)		12.1	2.9		8.2	4.4		12.6	1.7	
	Gender	Male	1466	(41.1)		12.4	2.0	*	9.1	3.9		12.8	1.2	**
		Female	2098	(58.9)		12.3	1.7		9.3	3.6		12.7	1.0	
	Body mass index (BMI)	<21.5	1150	(33.5)		12.3	2.0		9.1	3.8	*	12.7	1.1	
		≥21.5	2284	(66.5)		12.4	1.7		9.4	3.5		12.7	1.1	
	History of pneumonia within 1 year	presence	62	(1.7)		13.0	1.0	**	8.0	4.6	*	13.1	0.9	**
		absence	3502	(98.3)		12.4	1.8		9.2	3.7		12.7	1.1	
	Current medical treatment													
	diabetes	Yes	437	(12.3)		13.1	1.0	**	9.1	4.0		13.2	1.0	**
		No	3127	(87.7)		12.3	1.9		9.2	3.7		12.7	1.1	
	stroke	Yes	49	(1.4)		12.8	0.8		9.2	3.9		13.0	0.7	
		No	3515	(98.6)		12.4	1.8		9.2	3.7		12.7	1.1	
	heart disease	Yes	357	(10.0)		13.1	0.8	**	9.3	3.7		13.2	0.8	**
		No	3207	(90.0)		12.3	1.9		9.2	3.7		12.7	1.1	
	cancer	Yes	139	(3.9)		13.4	1.5	**	9.7	3.4		13.6	1.0	**
		No	3425	(96.1)		12.3	1.8		9.2	3.7		12.7	1.1	
	Difficulties in eating tough foods	Yes	933	(26.2)		12.5	1.7		9.4	3.8	*	12.8	1.0	**
		No	2631	(73.8)		12.3	1.9		9.1	3.7		12.7	1.1	
	Difficulties in swallowing tea or soup	Yes	770	(21.6)		12.5	1.9		9.4	3.5		12.8	1.1	**
		No	2794	(78.4)		12.3	1.8		9.2	3.8		12.7	1.1	
	Subjective oral frailty symptom^c^	One or more	1410	(39.6)		12.4	1.8		9.4	3.7		12.8	1.0	**
		None	2154	(60.4)		12.3	1.8		9.1	3.7		12.7	1.1	
Oral examination														
	The number of teeth present	20 teeth or more	2163	(60.7)		12.4	1.7		9.3	3.6		12.7	1.0	
		19 teeth or less	1400	(39.3)		12.3	2.0		9.2	3.9		12.7	1.1	
	Accumulation of tongue coating	None or slight	2232	(62.9)		12.3	1.9	**	9.3	3.6		12.7	1.1	**
		Moderate or severe	1316	(37.1)		12.5	1.7		9.1	3.9		12.8	1.1	
	Dry mouth	Normal	2474	(69.4)		12.4	1.8		9.2	3.7		12.7	1.1	*
		Mild, moderate, or severe	1025	(28.8)		12.4	1.9		9.1	3.8		12.8	1.1	
	CPI (Periodontal Pocket)	CPI = 0	2479	(81.1)		12.4	1.8		8.9	3.9	**	12.7	1.1	
		CPI ≥ 1	578	(18.9)		12.4	1.8		9.4	3.6		12.7	1.1	

^a^ Each cumulative value of expenditure (Yen) was performed natural logarithm conversion [log(expenditure +1)].

^b^ Student *t*‐test or variance analysis. *p < .05; **p < .01.

^c^ It shows the result of reaggregating “Difficulties in eating tough foods” and “Difficulties in swallowing tea or soup.”

### 2.4 Items of oral health examination

The items of oral health examination used in this study were as follows: the number of present teeth (19 teeth or less/20 teeth or more), accumulation of tongue coating (none or slight/moderate or severe), and dry mouth (normal/mild, moderate, or severe) according to the classification (Kakinoki et al., 2004). The periodontal pocket was measured by CPI (Community Periodontal Index) using the criteria of the 2013 revision of the method (World Health Organization, 2013). The tooth with no periodontal pocket was defined as “CPI = 0,” and the tooth with a shallow periodontal pocket of 4–5 mm or a deep periodontal pocket of 6 mm or more was defined as “CPI ≥ 1” and used for target items.

### 2.5 Statistical analysis

Before expenditure analysis, the cumulative value of annual medical expenditures, dental expenditures, and total expenditures was performed by natural logarithm conversion [log (expenditure +1)] because they were irregular distributions and those include values of 0. By performing the logarithmic conversion, it is possible to have approximate data that does not have a normal distribution with large variations to a normal distribution (Okamoto, 2010). Regarding the cross‐sectional study, the difference of three kinds of expenditures among the category in the questionnaire items or oral examination items were analyzed by student *t*‐test or the variance analysis. In addition, the difference in cumulative medical expenditures for 6 years in participants with adverse health outcomes or in healthy participants was analyzed by student *t*‐test.

The multiple regression analysis for 538 participants was also performed using the cumulative value for 6‐year expenditures as the outcome in the longitudinal study. The objective variable was medical, dental, or total expenditures in each participant and the explanatory variables were items of subjective oral frailty, the number of present teeth, accumulation of tongue coating, dry mouth, and CPI. The items of age, gender, BMI, history of pneumonia, and current treatment of diabetes, stroke, heart disease, and cancer were used as regulators injected in this analysis.

The occurrence of disability (support/long‐term care certification) or mortality during the 6‐year observation was used as the outcome, then the association between subjective oral frailty and the cumulative incidence of disability or mortality was evaluated by the log‐rank test in the Kaplan‐Meier analysis. In addition, by the cox proportional hazard analysis, the cumulative incidence of disability or mortality for 6 years was used as an objective variable, and items of oral frailty, the number of present teeth, accumulation of tongue coating, dry mouth, CPI (periodontal pocket) were used as explanatory variables. The items of age, gender, BMI, history of pneumonia, and current treatment of diabetes, stroke, heart disease, and cancer were used as regulators injected in this analysis.

In this study, the variance inflation factors (VIF), which is an index for detecting multiple co‐linearity, were all less than 10. It indicated that the possibility of multicollinearity among the items used as explanatory variables was low. SPSS Statistics 26 (IBM Japan) was used for statistical analysis, and the statistical significance level was set to less than 0.05.

### 2.6 Ethics

Regarding obtaining consent from each participant to join this study, we prepared a check box for consent to provide information on the oral health examination form including the questionnaire survey. The Ethics Committee of Tokushima University Hospital approved this study (Protocol approval number 2599‐2).

## 3 RESULTS

### 3.1 Characteristics of the participants and medical and dental expenditure

Table 1 shows the characteristics of the participants and the expenditure. Among 3564 participants, 41.1% were male and 58.9% were female. The significant differences of items in the category of medical expenditure were as follows: age—85 years old; gender—male; history of pneumonia—yes; current treatment—diabetes, heart disease, cancer, and accumulation of tongue coating. Total expenditure showed the same tendency in addition to difficulties in eating tough foods, difficulties in swallowing tea or soup, and dry mouth; mild, moderate, or severe. On the other hand, the significant differences of items in the category of dental expenditure were as follows: age—75 years old; gender—male, BMI: > 21.5; history of pneumonia—no; difficulties in eating tough foods—yes; and CPI; ≥ 1, which is slightly different from that of medical and total expenditure.

### 3.2 The longitudinal study regarding medical and dental expenditures

Table 2 shows the results of multiple regression analysis of the expenditures in the longitudinal study. The significant difference in item and category associated with medical, dental, and total expenditures was only subjective oral frailty.

**Table 2:** Multiple regression analysis^a^ for the factors related to medical, dental, and total expenditures by the longitudinal study.

					Collinearity statistics	
Dependent variables	Independent variables	β	95% confidence interval	*p*‐value	Tolerance	Variance inflation factors
Medical expenditures	Subjective oral frailty	.16	0.14–0.49	<.01	0.99	1.01
Dental expenditures	Subjective oral frailty	.10	0.07–1.36	<.05	0.99	1.02
Total expenditures	Subjective oral frailty	.18	0.16–0.46	<.01	0.99	1.01

^a^ Adjusted by gender, body mass index, history of pneumonia, current medical treatment of diabetes, stroke, heart disease, and cancer.

### 3.3 The longitudinal study regarding the occurrence of disability or mortality

**Figure 2:**
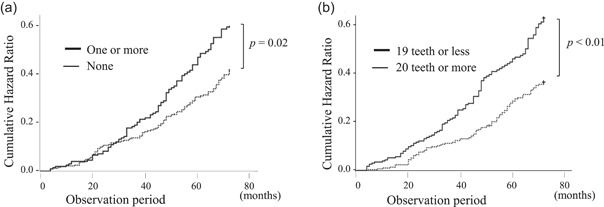
Kaplan–Meier analysis. (a) The subjective oral frailty. (b) The number of teeth present.

The participants enrolled in the longitudinal study for 6 years (72 months) were 538 and it was 7.4% of the residents of the same age. The occurrence of disability or mortality was 195 participants (certified long‐term care: 127; died: 68) and it was 36.2% of the total participants. Table 3 shows the results of the cox hazard analysis after adjusted factors, and Figure 2 shows the results of the cumulative hazard ratio during the observation periods. The cumulative incidence of disability or mortality increased significantly in items of subjective oral frailty; one or more (*p* = .02) and the number of remaining teeth; 19 teeth or less (*p* < .01).

**Table 3:** Association between oral condition and adverse health outcomes in the latter‐stage older adult.

	Model 1 (not adjusted)			Model 2 (adjusted)		
Items	HR	(95% CI)	*p*‐value^a^	HR	(95% CI)	*p*‐value^a^
Subjective oral frailty						
None	1.00	(ref)	.01	1.00	(ref)	.02
One or more	1.42	(1.08–1.88)		1.39	(1.05–1.84)	
Number of teeth present						
20 teeth or more	1.00	(ref)	<.01	1.00	(ref)	<.01
19 teeth or less	1.74	(1.32–2.29)		1.47	(1.10–1.95)	

*Note*: Model 2: adjusted by gender, body mass index, history of pneumonia, current medical treatment of diabetes, stroke, heart disease, and cancer.

^a^ Cox proportional hazard analysis; CI, confidence interval; HR, hazard ratio.

### 3.4 Association between the occurrence of adverse health outcomes and medical and dental expenditures

The difference in cumulative medical and dental expenditures in healthy participants and those with the occurrence of adverse health outcomes during the 6‐year period was analyzed. As shown in Table 4, medical expenses, dental expenses, and total medical expenses were significantly higher in participants with disability or mortality than that in healthy participants. Regarding dental expenses, however, contra‐versus results were obtained.

**Table 4:** The difference in cumulative medical, dental, and total expenditures for 6 years in healthy subjects or in subjects with adverse health outcomes.

				Medical expenditure^a^			Dental expenditure^a^			Total expenditure^a^		
Adverse health outcome		*n*	(%)	Mean	SD	*p*‐value^b^	Mean	SD	*p*‐value^b^	Mean	SD	*p*‐value^b^
None		343	(63.8)	14.5	1.2	**	11.3	3.5	**	14.7	0.8	**
Occurance		195	(36.2)	15.2	0.9		11.0	3.8		15.4	0.9	

^a^ Each cumulative value of expenditure (Yen) was performed natural logarithm conversion [log(expenditure +1)].

^b^ Student *t*‐test. **p < .01.

## 4 DISCUSSION

This longitudinal study revealed that there were relationships between increasing medical, dental, and total expenditures, and possessing oral frailty symptoms in the latter‐stage older adults at baseline. Furthermore, oral frailty symptoms were significantly related to the occurrence of disability or mortality for 6 years. This is the first report that relates subjective oral frailty in latter‐stage older adults to both subsequent increases in medical and dental expenditures and the occurrence of adverse health outcomes.

Oral functions in older adults are closely related to general nutritional status and quality of life. Several studies have demonstrated that difficulties in swallowing or decline of chewing function was associated with loss of appetite and diminished food diversity, and increased deterioration of general health condition through decreasing metabolic rate or malnutrition (Iwasaki, Kimura, Yoshihara, Ogawa, Yamaga, Takiguchi, et al., 2015, Iwasaki et al., 2016; Kikutani et al., 2013). It was also reported that patients with hypoalbuminemia had a higher incidence of complications and longer hospital stays. Serum albumin was the strongest clinical predictor of cost for hospitalized patients (Reilly et al., 1988).

Oral frailty in older adults is thought to be associated with social frailty, which includes the inability to speak well due to the deterioration of oral function and avoiding contact with people, leading to social isolation. It reported that there were significant associations between oral frailty and a decline in social function, physical function, and nutritional status (Hironaka et al., 2020). In addition, it was shown by path analysis that social frailty was directly related to oral frailty, and oral frailty was directly related to physical frailty.

Social isolation might cause health problems. It is reported that social interaction, such as communication with outsiders in addition to the family, played an important role in reducing medical expenditure for older adults (Sugisawa et al., 2009). Several review articles reported that consistent and compelling evidence linking a low quantity or quality of social ties with a host of conditions, including development and progression of cardiovascular disease, recurrent myocardial infarction, atherosclerosis, autonomic dysregulation, high blood pressure, cancer and delayed cancer recovery, and slower wound healing (Umberson & Karas Montez, 2010). In this study, oral frailty may have caused not only the deterioration of general conditions such as malnutrition but also social frailty, which may have affected spiraling medical and dental expenditure.

It was demonstrated that medical expenditure was significantly related to the subjective assessment of oral health in older adults (Harada et al., 2012). Regarding oral health, there was only an association between medical/total expenditure and the accumulation of tongue coating in this cross‐sectional study. The accumulation of tongue coating may be a risk factor for aspiration pneumonia, especially, in an edentulous older adult patient (Abe et al., 2008). Therefore, the accumulation of tongue coating may lead to an increase in medical expenditure. Michiwaki et al. reported that 20,000 people aged 70 and over in Japan are hospitalized for aspiration pneumonia every day, and the annual hospitalization costs are about 445 billion yen, and this amount corresponded to 3.1% of the total medical hospitalization expenditure and 16.6% of the dental expenditure (Michiwaki & Sumi, 2014).

It was also reported that periodontitis based on the periodontal inflamed surface area (PISA) might be an important predictor for excess medical expenditure among older Japanese people (Sato et al., 2016). Periodontal inflammation might be the key underlying factor for the deleterious effects of periodontitis on multiple organ systems (Kuo et al., 2008). In this study, periodontal pockets of 4 mm or more were related to dental expenditure but they did not relate to medical or total expenditure. In future studies, we plan to evaluate proper periodontal index, which includes inflammation status such as PISA.

On the other hand, no association between medical and dental costs and oral frailty symptoms was found in the cross‐sectional studies. Regarding the results of medical expenditure in a single‐year survey when compared to the longitudinal study, it may have been affected by the quality and the number of outpatients visiting the hospital or clinic. Therefore, the relationship between medical expenditure and oral frailty symptoms may be less likely to appear.

It was reported that oral functional limitations associated with tooth loss might lead to avoidance of certain foods, such as vegetables, nuts, and fruits, which can lead to decreased dietary diversity, including vitamins and minerals, among older Japanese women (Iwasaki, Kimura, Yoshihara, Ogawa, Yamaga, Wada, et al., 2015). There was also an association between the “presence of fewer than 19 teeth” and the “occurrence of disability or mortality” in our longitudinal study.

On the other hand, patients with more teeth spent less on medical care. In fact, medical care expenditure increased by 2.4% per tooth loss in patients aged 50's (Tsuneishi et al., 2017). There was no association between the number of teeth and medical and dental expenditure in our study. It was also reported that female participants with less than 10 functional teeth and without dentures showed a significantly higher mortality rate than those with dentures (Fukai et al., 2008). It will be necessary to investigate the use of dentures and the state of occlusion in future studies.

It seems that preventing the need for long‐term care and extending healthy life expectancy will lead to a reduction in medical expenditure. The medical expenditures of participants who had adverse health outcomes were significantly higher than those who did not, whereas the opposite result was obtained regarding dental expenditures. It is possible that participants who died or were admitted to older adult institutions due to the need for long‐term care during the observation period were unable to visit a dental clinic, which may lead to reduced dental expenditures.

The participants who received oral health examinations in a dental clinic were enrolled in this study, and therefore, there is a possibility that the target population was limited to those who were healthy enough to visit a dental clinic. To clarify the characteristics of the participants in this study, we obtained the data regarding the 2016 annual cumulative expenditures of all residents aged 75, 80, 85, and 90 years in Tokushima City from the Tokushima Association Responsible for Operation of the Health‐Care System for the Latter‐Stage Older Adult, and a supplemental table was created for the consideration. As shown in Supplemental Table 1, the participants aged 75, 80, 85, and 90 years were 8.7%, 8.0%, 3.6%, and 0.9%, respectively. It revealed almost no difference in average cumulative medical and total expenditures at the 75 and 80 age groups between all residents and participants in this study.

Supporting Information: Table S2 shows the results of multiple regression analysis of the expenditures in the 75 and 80 age group which is 85.3% of the total participants. The significant difference between the item and category associated with medical and total expenditures was only subjective oral frailty as well as Table 2. Supporting Information: Table S3 shows the results of the difference in cumulative medical, dental, and total expenditures for 6 years in healthy participants aged in the 75 and 80 age group or in participants with adverse health outcomes. The medical expenses and total expenses were significantly higher in participants with disability or mortality than that in healthy participants. Table 2 in the results and Supporting Information: Table S2 shows the same tendency regarding the medical expenses and total expenses, as well as Table 4 in the results and Supporting Information: Table S3. These results suggest that the participants in this study might be considered a representative group of residents in Tokushima City and the results of the analysis in this study were sufficient for the discussion of the relationship between subjective oral frailty and medical expenditures.

There are limitations to this study. The first point is that the individual medical history related to general diseases was derived only from answers to the questionnaire completed by the participants themselves. So, there was a possibility for a potential bias regarding the general condition. Hiratsuka et al. have reported based on data from 43,740 specific health checkup participants that the medication for low blood pressure, blood sugar, or cholesterol levels was associated with increases in medical expenditures (Hiratsuka et al., 2017). In addition, physical activity, fast walking, good sleep patterns, and health guidance were associated with decreases in medical expenditures. This report suggests that the parameters of health status in our study may be inadequate for analysis because the items did not include the status of medication and health habits of the participants.

In addition, KDB did not provide the details of the breakdown of medical expenditures such as what kinds of diseases, or whether participants were inpatient or outpatient. Therefore, it was unclear what kind of general disease was related to subjective oral frailty.

This study showed that subjective oral frailty in latter‐stage older adults is related to adverse health outcomes and an increase in medical and dental expenditures. From the perspective of extending healthy life expectancy and reducing medical expenditures, it is necessary to make many people aware of oral frailty and to strengthen measures against oral frailty in the future. Therefore, it is expected that the new findings obtained in this study will be utilized as basic data for promoting measures of oral health.

## 5 CONCLUSIONS

It was revealed that among latter‐stage older adults, individuals without certified nursing care and those with subjective oral frailty at baseline had significantly higher medical, dental, and total expenditures from this 6‐year longitudinal study. In addition, those with subjective oral frailty or with less than 19 teeth were shown to have a higher possibility for the occurrence of disability or mortality.

## AUTHOR CONTRIBUTIONS

Daisuke Hinode designed, coordinated, and performed the study and drafted the paper. Tokiko Doi, Makoto Fukui, and Masami Yoshioka designed, performed the statistical analysis, and drafted the paper. Yoshifumi Okamoto, Manabu Shimomura, Kimi Matsumoto, and Miwa Matsuyama coordinated and drafted the paper. All authors reviewed the paper critically for content and approved it for submission.

## CONFLICT OF INTEREST STATEMENT

The authors declare no conflict of interest.

## Supporting information

Supplementary information.

## Data Availability

The authors confirm that data supporting the findings of this study are available within the article. Data will be shared on reasonable request to the corresponding author.
